# *In-Situ* Spectro-Electrochemistry of Conductive Polymers Using Plasmonics to Reveal Doping Mechanisms

**DOI:** 10.1021/acsnano.2c09081

**Published:** 2022-12-05

**Authors:** Jialong Peng, Qianqi Lin, Tamás Földes, Hyeon-Ho Jeong, Yuling Xiong, Charalampos Pitsalidis, George G. Malliaras, Edina Rosta, Jeremy J. Baumberg

**Affiliations:** † NanoPhotonics Centre, Cavendish Laboratory, Department of Physics, University of Cambridge, Cambridge CB30HE, U.K.; ‡ Department of Physics and Astronomy, University College London, London WC1E 6BT, U.K.; § Department of Chemical Engineering and Biotechnology, University of Cambridge, Cambridge CB30AS, U.K.; ∥ Electrical Engineering Division, Department of Engineering, University of Cambridge, Cambridge CB30FA, U.K.

**Keywords:** conductive polymers, redox, doping mechanism, spectro-electrochemistry, plasmonics, nanoparticle, surface-enhanced Raman scattering

## Abstract

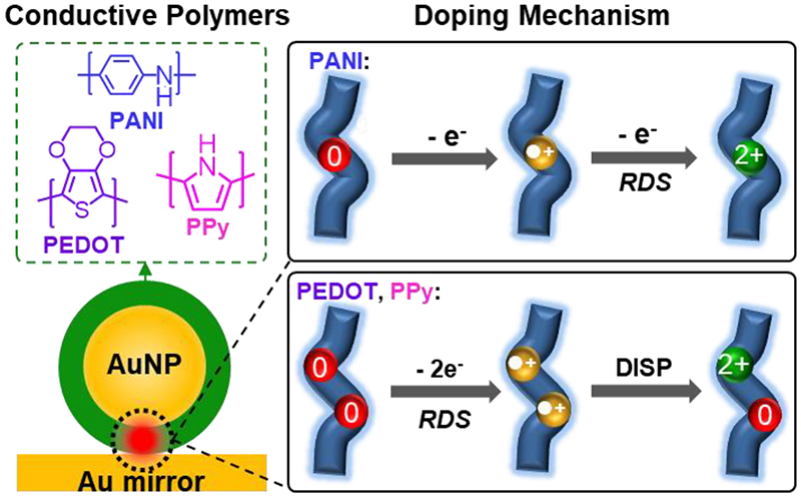

Conducting polymers are a key component for developing wearable organic electronics, but tracking their redox processes at the nanoscale to understand their doping mechanism remains challenging. Here we present an *in-situ* spectro-electrochemical technique to observe redox dynamics of conductive polymers in an extremely localized volume (<100 nm^3^). Plasmonic nanoparticles encapsulated by thin shells of different conductive polymers provide actively tuned scattering color through switching their refractive index. Surface-enhanced Raman scattering in combination with cyclic voltammetry enables detailed studies of the redox/doping process. Our data intriguingly show that the doping mechanism varies with polymer conductivity: a disproportionation mechanism dominates in more conductive polymers, while sequential electron transfer prevails in less conductive polymers.

Conductive polymer thin films underpin advances in organic electronics due to their low-cost fabrication compared to silicon counterparts and their mechanical flexibility compatible with foldable devices.^1^ The reversible doping/dedoping of conductive polymers is the basis of organic thin-film transistors,^2,3^ sensors,^4^ and displays.^5,6^ Doping transfers electrons in/out (i.e., reduction/oxidation) of the neutral conductive polymers, creating negative/positive charge carriers.^7,8^ Two-electron transfers are involved in generating polarons or bipolarons. The former contain monoradical ions (P^●–^ or P^●+^), and the latter contain di-anions/cations (P^2–^ or P^2+^). Although many techniques have been used to characterize the doping/redox process, conflicting conclusions often arise from the difficulty in obtaining a well-defined electrochemical response from the polymers, and it is unclear why short-lived polaron intermediates are sometimes observed but sometimes not.^9−12^ Understanding of the doping/redox mechanism is thus scarce, limiting development of widespread polymer-based applications.

A promising technique to address this challenge integrates such polymers into nanocavities, where their optical and vibrational properties can be interrogated in real time. By using plasmonic nanocavities which support optical hotspots in the nanogap between coinage metal components, the light can be confined to nanoscale volumes of the polymer, while simultaneously electrons are transported only a few nanometers from these metal contacts (hence, this is rapid). We thus coat thin films of conductive polymers around gold nanoparticles (Au NPs) which are drop-cast onto a gold substrate, forming an electrochromic nanoparticle-on-mirror (*e*NPoM, Figure 1a) geometry. Such a plasmonic geometry has a well-defined electrochemical response^5,6^ and yields fast-switching color dynamics at the single-nanoparticle level. However, dark-field spectroscopy gives too little information for mechanistic studies seeking to identify the intermediates involved in the complex two-electron transfers. Surface-enhanced Raman spectroscopy (SERS), in combination with cyclic voltammetry, is a powerful technique to uncover structural fingerprints at the few-molecule level during redox transitions.^13^ Exploiting the NPoM geometry which supports highly confined optical fields in the plasmonic hotspot gives a vibrational Raman scattering enhancement of >10^4^. This technique thus allows *in situ* study of the thin-film conductive polymer redox in real time to reveal features of the doping mechanism.

**Figure 1:**
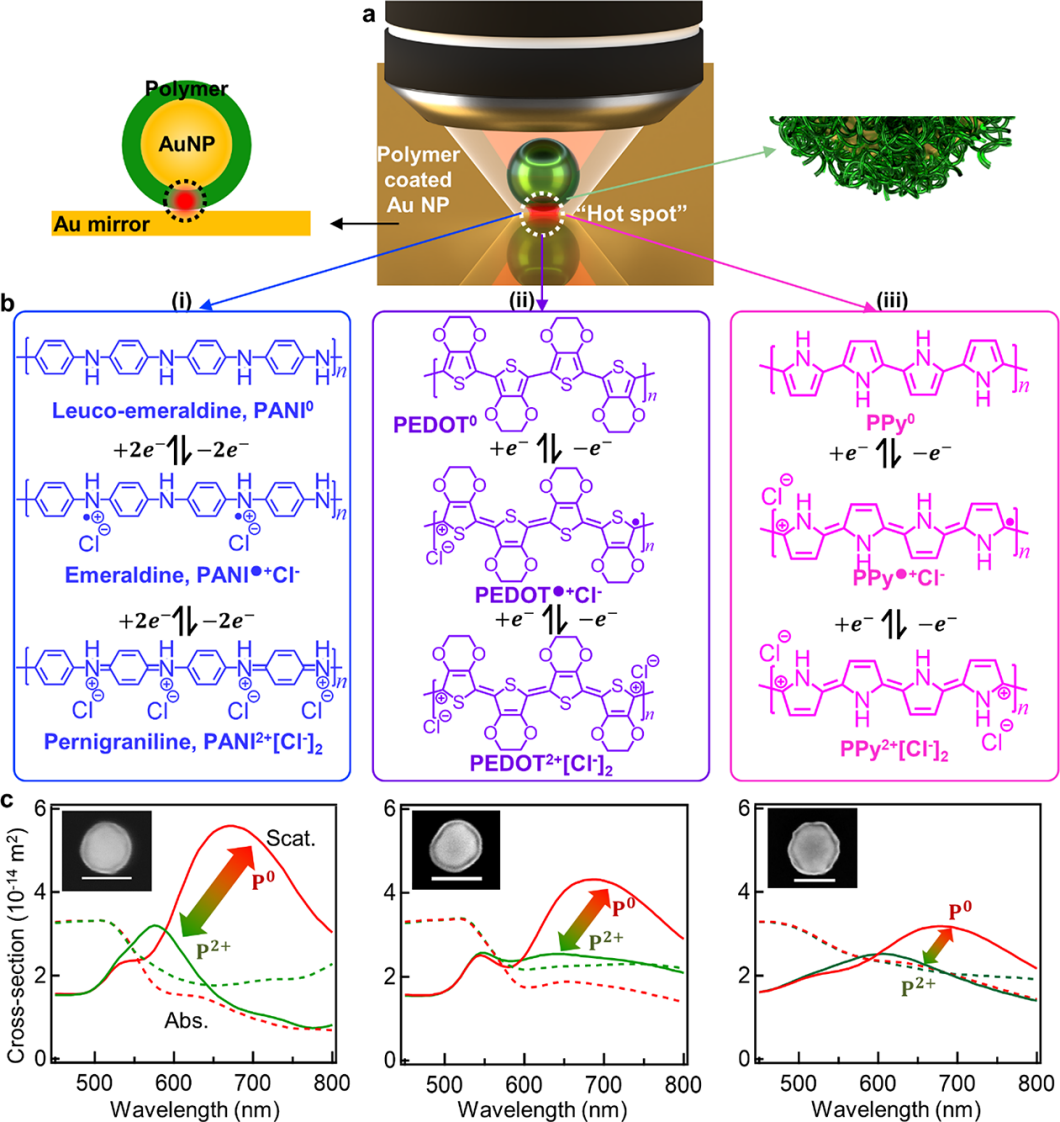
Conductive polymer redox in plasmonic hotspots. (a) Schematic of electrochromic nanoparticle-on-mirror (*e*NPoM). Each gold nanoparticle (Au NP) is coated with (i) polyaniline (Au@PANI), (ii) poly-3,4-ethylenedioxythiophene (Au@PEDOT), or (iii) polypyrrole (Au@PPy). Insets: cross-section of *e*NPoM (left) and zoom-in view of coated nanoparticle (right). (b) Redox polymer reactions (P^0^, reduced; P^●+^, intermediate; P^2+^, oxidized), represented in tetramers with *n* repeat units. (c) Finite-difference time-domain (FDTD) simulated absorption (dashed) and scattering (line) spectra of individual *e*NPoMs for P^0^ and P^2+^ states. Insets: scanning electron microscopic (SEM) images showing the core–shell structure. Au NP diameter is 80 nm, polymer thickness is 20 nm, and scale bar is 100 nm.

We initially demonstrate reversible color switching of such *e*NPoMs using dark-field (DF) spectroscopy on Au NPs coated with either polyaniline (Au@PANI), poly-3,4-ethylenedioxythiophene (Au@PEDOT), or polypyrrole (Au@PPy). These are deposited around Au NPs in solution using surfactant-assisted chemical oxidative polymerization (Figure 1c insets) to 20 nm thickness for all data shown here (see Methods). The observed color dynamics in plasmonic scattering (Figure 1c) is a result of redox-induced changes in the molecular electronic transitions (Figure 1b), leading to changes in absorption and refractive index.^14−16^*In-situ* surface-enhanced Raman spectroscopy is then used to probe the molecular vibronic changes during redox. The tight optical field confinement within individual plasmonic nanocavities gives high signal-to-noise ratios, allowing a spectro-electrochemical study of conductive polymers during redox down to volumes ∼(20 nm)^3^.

## Results and Discussion

### *e*NPoM Optical Switching

**Figure 2:**
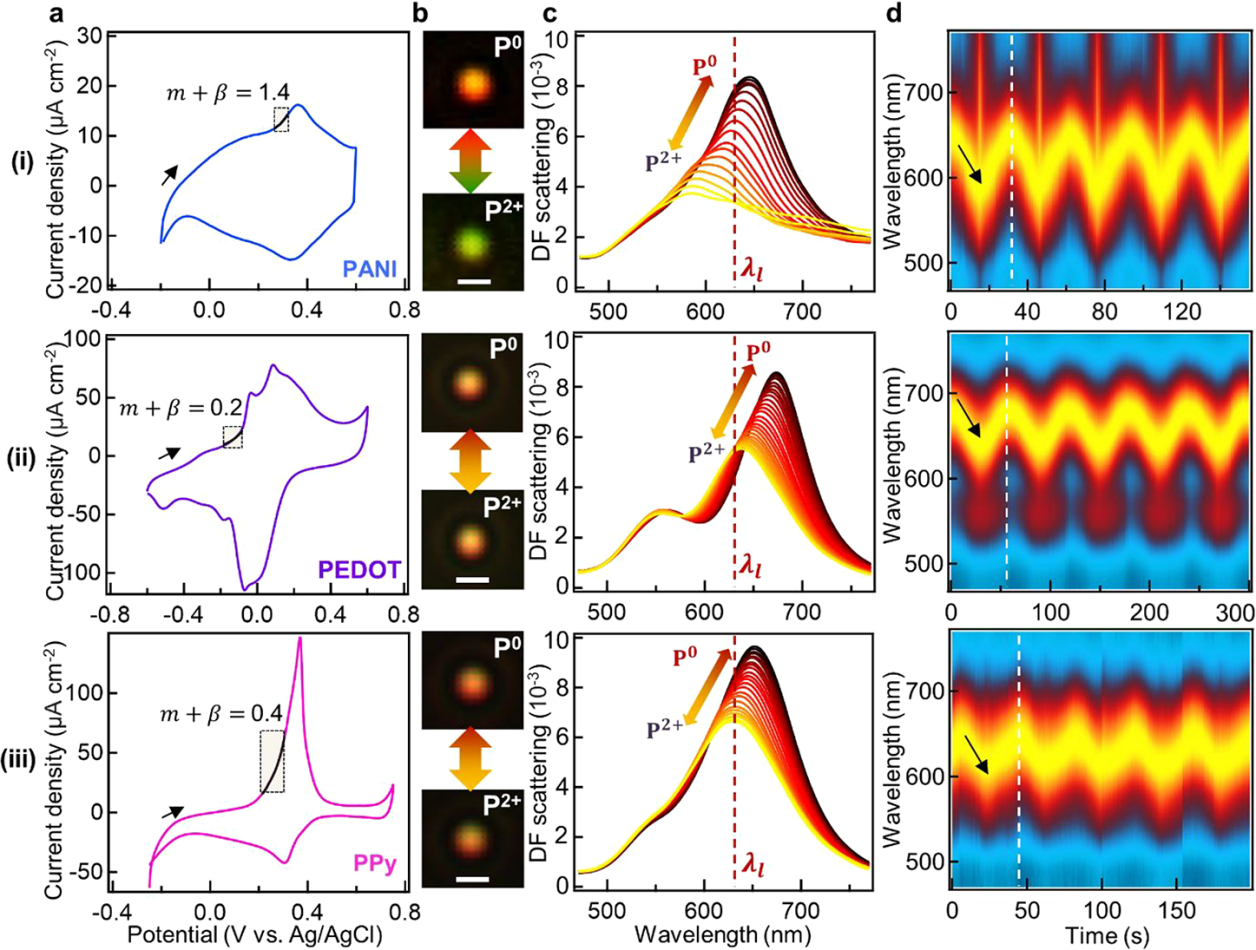
Optical switching of *e*NPoMs. (i) Au@PANI for ramped voltages −0.2 ↔ 0.6 V, (ii) Au@PEDOT for −0.6 ↔ 0.6 V, and (iii) Au@PPy for −0.25 ↔ 0.75 V. (a) Cyclic voltammetry (CV) with apparent transfer coefficients (*m* + β, see background non-Faradaic capacitive current in SI Figure S5, and Tafel plots in Figure S6) ranging from 0 to 2, showing the transition state is reactant-like (P^0^) or product-like (P^2+^). Scan rate 50 mV s^–1^. (b) Dark-field (DF) scattering images, scale bar 1 μm. (c) DF scattering spectra of single *e*NPoMs as potential applied. Dashed *λ*_l_ = 633 nm is laser wavelength used for surface-enhanced Raman spectroscopy (SERS, see below). (d) Time-series normalized DF scattering spectra from single *e*NPoMs over 5 cycles.

To track the conductive polymer redox optically, customized spectro-electrochemical cells with three electrodes are used to control the shell polymer redox state (see Methods and Supporting Information (SI) Figure S1). All three polymers can undergo 2*e*^–^ transfers resulting in three redox states (Figure 1b), with appropriate electrolytes (see Methods). Finite-difference time-domain (FDTD) simulations of the optical scattering spectra (Figure 1c and SI Figure S2) using optical permittivities from the literature^17−20^ show that the long-wavelength plasmonic coupled mode for all three polymer-coated *e*NPoMs will blue-shift upon oxidation. This is confirmed by the experimental DF scattering spectra measured vs applied potential (Figure 2c). The oxidation of PANI gives a coupled-mode peak wavelength shift from around 645 to 578 nm, for PEDOT from 670 to 635 nm, and for PPy from 630 to 610 nm. Highly stable and reversible optical switching is observed (Figure 2d). Both PPy and PEDOT tune over narrower wavelength ranges than PANI, making their color dynamics harder to recognize by eye from their DF images (Figure 2b and SI Figure S4). Although not as good as Au@PANI in color dynamics for display applications,^5,6^ Au@PPy and Au@PEDOT have been used in switching devices^21,22^ as their absorption varies with their different redox states. Further FDTD simulations from different nanostructure configurations show the NPoM geometry gives consistent near-field enhancements in the gap, providing reliability for quantitative analysis of these polymers (SI Figure S3).

### Polymer Redox Mechanism

To understand the charge transfer process during polymer redox, the recorded cyclic voltammetry (CV, Figure 2a) is analyzed in two respects. Thermodynamically, only one voltametric peak is observed for the 2*e*^–^ process in all three polymers, showing the potentials for the two electronic transitions are “potential inverted”;^23^ i.e., oxidation of P^●+^ is easier than oxidation of P^0^. Note this also applies to Au@PEDOT, although a set of small prepeaks at ∼ −0.2 V are observed due to the strongly trapped/released dopant counterions.^7,24^ Whether the P^●+^ intermediate is then observable will be governed by the rate of electron transfer (i.e., kinetics). Kinetically, the oxidative current is then plotted on a log scale (SI Figure S6, Tafel analysis^25^) to determine the transfer coefficients (*m* + β). For a multielectron transfer process, it is possible to extract from Tafel plots an apparent transfer coefficient to determine the rate-determining step.^23,25,26^ Prior to this Tafel analysis, a background correction is used to remove the non-Faradaic contribution to the current from capacitance (SI Figure S5). The local potential in the NPoM gap has been previously calibrated by the vibrational Stark effect.^13^ This shows that after non-Faradaic capacitive charging, the potential drops efficiently within 1 nm from the electrode surface, so Faradaic electron transfer happens readily between the Au mirror and molecular layer.

**Scheme 1:**
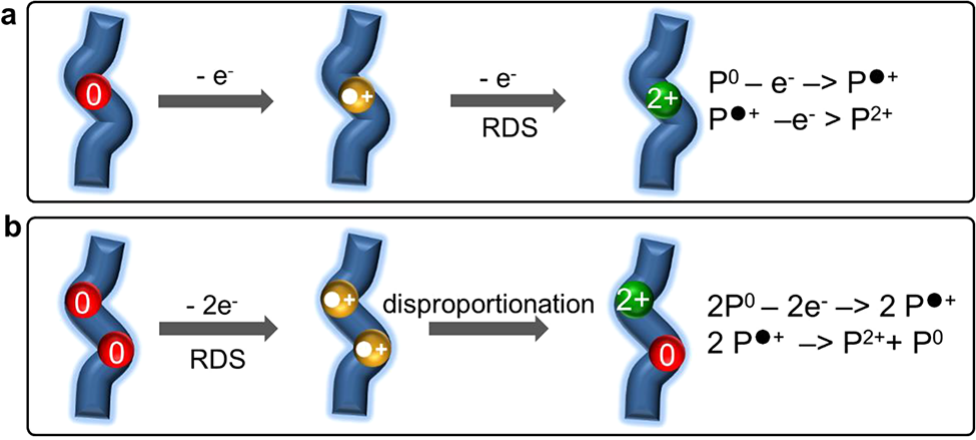
Mechanism of Conductive Polymer Redox Two pathways for oxidative doping: (a) Consecutive two-step electron transfer: P^0^ → P^●+^ → P^2+^ by losing two electrons step-wise. (b) Disproportionation-type electron transfer: P^0^ → P^●+^ by losing one electron; then two P^●+^ rapidly combine to form a P^2+^ without extra electron transfers. RDS: rate-determining step.

The apparent transfer coefficients are indicators for the rate-determining step (RDS), which is the (*m* + 1)^th^ electron transfer. As (*m* + β) = 1.4 for PANI, the second electron loss is the RDS (i.e., oxidation of P^●+^). By contrast, (*m* + β) = 0.2 for PEDOT and 0.4 for PPy, showing the first electron loss is the RDS (i.e., oxidation of P^0^). Two different types of electron-transfer mechanism thus occur during the redox transition (Scheme 1). For PANI, predominantly consecutive two-electron loss takes place stepwise, so all three redox states should be observed during the reaction. For PEDOT and PPy, after the first electron loss, P^●+^ quickly combines with another P^●+^ and disproportionates into P^2+^ and P^0^,^26^ so the P^●+^ intermediate is not observable.

Disproportionation becomes favorable when no extra energy is required to drive further electron transfer, and the process is triggered when the P^●+^ intermediate is stabilized.^26^ Since electrons are more easily delocalized in PEDOT^●+^ and PPy^●+^ which possess more resonance structures (SI Figure S7a), they are more stable than PANI^●+^. This is also reflected in the conductivity of ∼10^3^ S cm^–1^ for PEDOT^●+^ and PPy^●+^, which is much higher than that for PANI^●+^ of ∼30 S cm^–1^.^27^ The lower conductivity of PANI is also evidenced by the higher capacitive background in CV (Figure 2a and SI Figure S5). Therefore, the disproportionation-type mechanism seems to predominate in more conductive polymers, while stepwise electron transfer prevails in less conductive polymers.

### Tracking Dynamics and Detecting Reaction Intermediates

**Figure 3:**
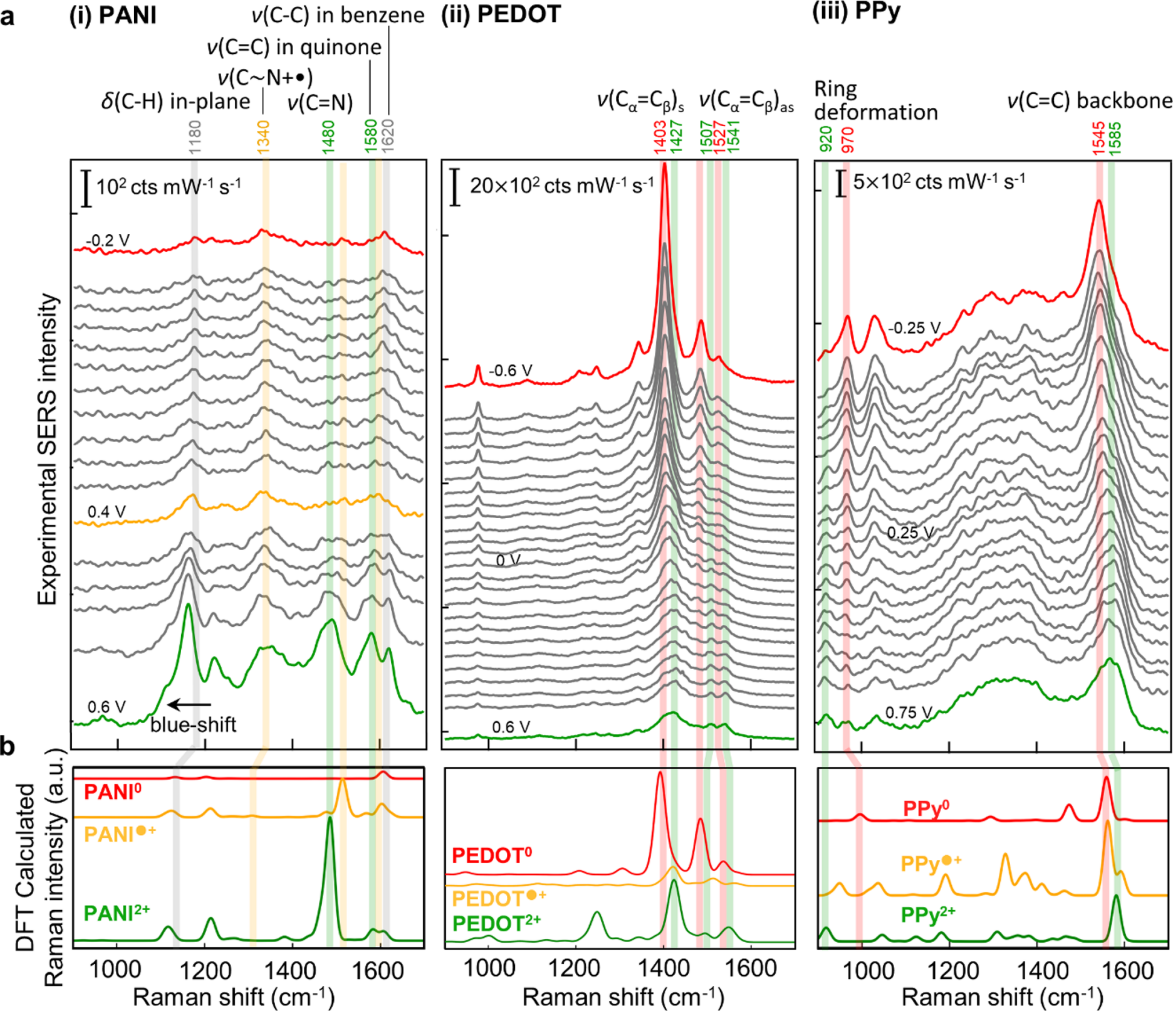
Raman evolution during redox. (a) Experimental SERS spectra vs voltage (top to bottom) as in Figure 2a. (b) DFT calculated Raman spectra. Red: P^0^, orange: P^●+^, green: P^2+^. Vibrational modes relevant to oxidation are highlighted in orange and green; persistent modes of reduced state highlighted in red; modes seen in all three redox states are highlighted in gray.

To examine further the polymer redox mechanism suggested by electrochemical measurements, *in situ* SERS spectra are recorded in real time vs applied potentials for the three *e*NPoM types (Figure 3a). The results are all comparable on different *e*NPoMs of the same type, showing consistency in the polymer shell coating and gap sizes. A 633 nm laser is used to provide efficiently outcoupled SERS spectra tuned near the plasmon resonance (Figure 2c), with a high signal-to-noise ratio using 1 s integration times for low powers of 100 μW (see Methods). We note higher powers easily induce photochemical changes of metal and molecule. DFT Raman calculations are performed to simulate the SERS bands for the three polymers in different redox states (Figure 3b). Comparison of computational spectra with experimental SERS facilitates assignment of distinct SERS bands to corresponding vibrational modes (see top of Figure 3a, SI Table S1). Compared to reference literature where polymers were studied in bulk, the main peaks observed here arise from polymers confined in plasmonic hotspots and show slight blue-shifts of a few cm^–1^, indicating that the polymers are possibly more crystalline or have longer conjugation lengths than their bulk counterparts,^28−30^ offering an interesting scope for future study on polymer morphology (see below).

**Figure 4:**
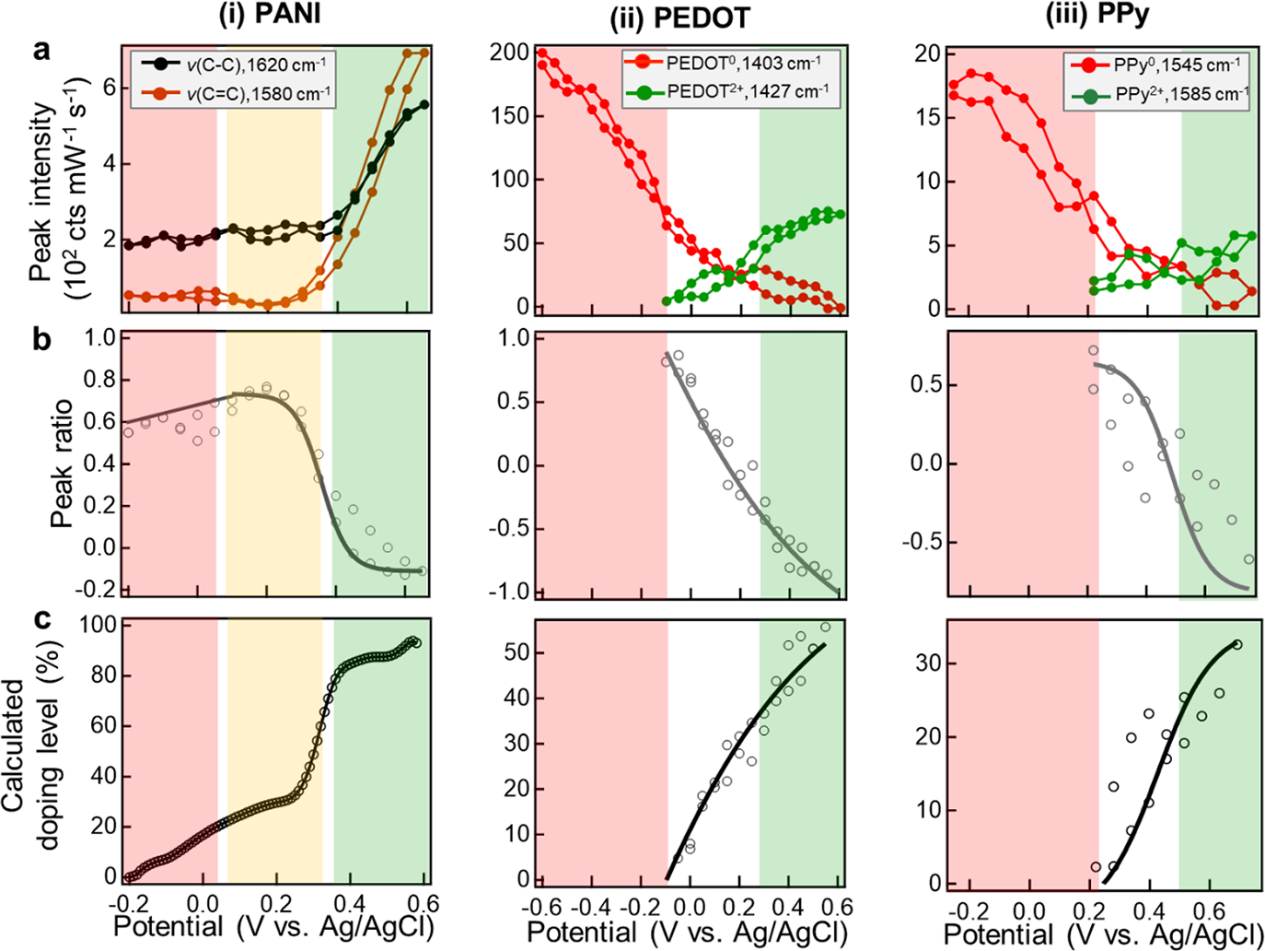
Analysis of SERS dynamics for tracking redox. (a) Extracted peak intensities vs applied potential of (i) ν(C–C) at 1620 cm^–1^ and ν(C=C) at 1580 cm^–1^ for PANI, (ii) symmetric ν(C_α_=C_β_) for PEDOT^0^ at 1403 cm^–1^ and PEDOT^2+^ at 1427 cm^–1^, and (iii) ν(C=C) for PPy^0^ at 1545 cm^–1^ and PPy^2+^ at 1585 cm^–1^. (b) Fractional ratio of the two components, defined as (*I*_re_ – *I*_ox_)/(*I*_re_ + *I*_ox_) at each V. (c) Calculated doping level vs applied voltage. The red/yellow/green shaded regions correspond to the dominance of P^0^/P^1+^/P^2+^ states from cyclic voltammograms in panel b, where region boundaries are defined by the onset and completion potentials of the oxidation peak. Solid lines are fits to sigmoid functions.

For PANI (Figure 3a (i)), three sets of SERS band changes are distinguished during the redox cycle. First, a set of bands at 1340 and 1480 cm^–1^ are associated with ν(C–N^+•^) and ν(C=N), respectively. While the latter is characteristic for PANI^2+^, the former arises from an intermediate bond (resonant between a single and a double bond, see SI Figure S7a i) linked to the protonated nitrogen atoms in PANI^●+^.^31,32^ Spectroscopic observation of PANI^●+^ aligns with the mechanism where consecutive two-step electron transfer occurs (see above, Scheme 1a). Second, a set of bands at 1580 and 1620 cm^–1^ correspond respectively to ν(C=C) in quinone rings from PANI^2+^, and ν(C–C) in benzene rings from all three redox states. Extracting their intensities shows absolute changes of the two bands with voltage (Figure 4a (i)), while their intensity ratio shows how the redox processes compare to the cyclic voltammetry (Figure 4b (i)). Most evident is that the intensity of all SERS bands of PANI increases upon oxidation (Figure 4a (i) and SI Figure S8a), in contrast to PPy and PEDOT (Figure 4a (ii, iii), discussed below). This is attributed to the blue-shifting molecular absorption of PANI with oxidation from around 890 to 750 nm,^33^ becoming closer to the excitation wavelength 633 nm, as well as the larger Raman cross sections given by the polarizable conjugated backbone from proton doping and dedoping (SI Figure S7b).^34^ This enhances SERS emission from oxidized PANI which dominates the spectra and makes it hard to discern changes in reduced PANI. In contrast, the molecular absorption of PPy red-shifts from 360 to 490 nm with oxidation,^35^ and PEDOT red-shifts from around 620 to 1600 nm with oxidation.^36^ Use of shorter wavelength lasers (488 or 532 nm) might help the detection of the reduced species, but plasmonic heating by gold interband absorption damages polymers in the gap during continuous measurement.^37,38^ Third, the band at ∼1180 cm^–1^ is assignable to the in-plane δ(C–H) associated with ring deformation throughout all three redox states. Although the intensity of this band is resonantly enhanced upon oxidation (SI Figure S8a), it also blue-shifts linearly with the applied potential (Figure S8b). Likely, δ(C–H) does not track redox because it is a pendant mode away from the polymer chain and less influenced by charge variations on the backbone compared with the other modes discussed above.^32^

For PEDOT (Figure 3ii), the most intense change in the SERS spectra is a band at 1403 cm^–1^, which continuously broadens and red-shifts to 1427 cm^–1^ upon oxidation. These broad peaks are resolved into two components: the band at 1403 cm^–1^ is the symmetric ν(C_α_=C_β_) from PEDOT^0^, and the other band at 1427 cm^–1^ is from PEDOT^2+^.^39,40^ According to analysis of these two components (Figure 4a (ii)), the band from PEDOT^0^ (red) initially dominates but decreases in intensity upon oxidation, with only (5 ± 5)% remaining when oxidation is complete. Meanwhile, the band from PEDOT^2+^ (green) starts to appear at the onset potential around −0.1 V and shows the opposite trend increasing by (74 ± 16)%. Their ratio tracks the increasing proportion of PEDOT^2+^ to PEDOT^0^ (Figure 4b (ii)), directly indicating the oxidation level. The simultaneous observation of both bands at intermediate redox indicates the coexistence of PEDOT^0^ and PEDOT^2+^. The presence of both states is further confirmed by the splitting of the band at 1527 cm^–1^ into two bands at 1507 and 1541 cm^–1^, associated with the asymmetric ν(C_α_=C_β_) from PEDOT^2+^ and PEDOT^0^.^41−43^

For PPy (Figure 3 (iii)), the most intense band is located at 1545 cm^–1^, which broadens and red-shifts to 1585 cm^–1^ upon oxidation. This broad feature can again be decomposed into a pair of modes, assigned to the ν(C =C) of the backbone from PPy^0^ and PPy^2+^, respectively.^9,11^ Peak intensity analysis (Figure 4a,b (iii)) shows the band from PPy^0^ decreases by (67 ± 15)% upon oxidation, and the band from PPy^2+^ increases by (40 ± 22)%, demonstrating formation of PPy^2+^. Observation of both bands again implies that PPy^0^ and PPy^2+^ coexist. The same trend and coexistence are observed in another pair of bands at 970 and 920 cm^–1^, assigned to the ring deformation modes from PPy^0^ and PPy^2+^, respectively (SI Figure S8c).

Comparing the SERS peak intensity changes for all three polymers (Figure 4a), it is obvious that PEDOT^2+^ and PPy^2+^ appear as soon as the potential hits the onset of oxidation (−0.1 V and +0.2 V) from the cyclic voltammograms (Figure 2a), while PANI^2+^ does not appear until the oxidation peak potential (+0.4 V) is reached. This strongly confirms the two different mechanisms noted above: in the former case disproportionation-type oxidation occurs giving P^2+^ immediately at the onset potential, and in the latter case stepwise electron transfer takes place generating P^2+^ gradually upon complete oxidation. In addition, it is noticeable that PEDOT^0^ and PPy^0^ SERS intensities decrease even before the onset of oxidation (Figure 4a (ii,iii)). Rather than indicating a decrease in P^0^, this is likely due to changes of polymer polarizability resulting from the hydrogen evolution reaction (HER)^44^ at negative potentials for the pH 2 solutions used. At positive potentials, no HER occurs, so SERS intensities can be directly correlated with P^0^ concentration.

### Estimation of Doping Levels

For PEDOT and PPy, analysis of SERS ratios can be used to estimate the proportion of reduced and oxidized states, and thus to estimate quantitatively the doping (oxidation) level (Figure 4c), which cannot be easily resolved from voltammograms. This calculation is less precise for PANI because of its electronic-resonance-enhanced response noted above (SI Figure S7b), resulting in dominant surface-enhanced resonance Raman scattering of PANI^2+^, but it is still provided as a rough estimation. Previous bulk Raman measurements on chemically doped PEDOT samples^39^ have calibrated the ratio of the symmetric ν(C_α_=C_β_) peaks to the doping level *D*,
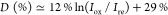
1where *I*_ox_ and *I*_re_ are intensities of P^2+^ and P^0^ SERS bands respectively, and this applies in the voltage range when *I*_ox_/*I*_re_ > 1. Using this calibration here suggests that the doping level of PEDOT increases from 0% at around −0.1 V to ∼50% when electrochemical oxidation completes (Figure 4c (ii)). This doping level is significantly higher than the literature reported values of chemical doping in bulk of <30%.^39^ We suggest that this is due to the use of only a thin 20 nm polymer coating on the convex Au surface which facilitates dopant ion diffusion.^45^ Assuming the backbone bonds in PPy (which are the same) follow the same doping dependence suggests that PPy doping increases to (40 ± 2)% when oxidation completes (Figure 4c (iii)), which has not been previously measured. One reason for the lower final doping achieved could be that PPy is susceptible to oxygen from the ambient environment, so oxidative degradation competes with the doping process.^46,47^ By contrast PEDOT has excellent air stability and higher conductivity,^48^ allowing for better switching and higher doping levels when oxidized. This accounts for the extensive use of PEDOT as a conductive material in applications.

The results show many subtle behaviors from polymer conformation and electrochemistry. For instance, additional effects are also observed when varying the polymer thickness and from choices of electrolyte.^49−51^ This enables many potential future directions to study how the polymer morphology, doping mechanism, polymer conductivity, and switching speed between different redox states, are influenced by different conditions. Intriguing prospects emerge when optical modes are further confined down to the sub-nm^3^ scale through “picocavities” formed by single adatoms, which can provide information on even single monomer redox events in a polymer chain. Such precision electrochemistry on the nanoscale is relevant to widespread applications, suggesting the approach shown here is of universal utility.

## Conclusions

We have demonstrated the concept of *e*NPoMs with three different nanothick conjugated conductive polymers, PPy, PEDOT, and PANI. Highly reproducible optical switching is observed for each using dark-field spectroscopy in real time. *In-situ* SERS is measured in combination with cyclic voltammetry and compared with density functional theory calculations to reveal the different redox/doping mechanisms for polymers in the gap. For PANI, consecutive two-step electron transfer takes place, although the strong electronic resonance in the oxidized state interferes with measuring precise doping levels. For PPy and PEDOT, disproportionation-type electron transfer occurs so that after the first electron is lost, the polaron formed combines with the neighboring polarons to form a bipolaron. The type of redox/doping mechanism is found to be correlated with polymer conductivity. This work shows that it is possible to measure doping levels quantitatively in real time using vibrational changes of the C=C backbone, in sample volumes of only a few (10 nm)^3^ corresponding to only a few hundred unit cells.

## Methods

### Polymer Coating on Gold Nanoparticles (Au NPs)

The coating of all three conductive polymers on Au NPs used surfactant-assisted chemical oxidative polymerization methods.^5,20,52^ 1.6 mL of the citrate-stabilized Au NPs (BBI Solutions) solution is concentrated, followed by removal of the supernatant, and then mixing with the monomer solution and the surfactant (here, sodium dodecyl sulfate, SDS). The monomer molecules are attracted to the surface of the Au NPs due to electrostatic forces and the polymerization takes place upon adding oxidant (here, ammonium persulfate, APS).

For polypyrrole-coated Au NPs (Au@PPy) or poly-3,4-ethylenedioxythiophene-coated Au NPs (Au@PEDOT), the concentrated Au NPs solution is mixed with 0.78 mL of 10 mM pyrrole or 14.5 mM 3,4-ethylenedioxythiophene aqueous solution, together with 0.145 mL of 40 mM SDS. After vigorously vortexing, 0.69 mL of 19.1 mM APS is added with 8.25 μL of 20 mM iron chloride (FeCl_3_) solution. FeCl_3_ acts as a catalyst here. For polyaniline-coated Au NPs (Au@PANI), 0.6 mL of 2 mM aniline is added to the Au NPs solution, together with 0.12 mL of 40 mM SDS, and then mixed with 0.6 mL of 2 mM APS in 10 mM hydrochloric acid (HCl). All these polymer-coated Au NPs solutions are incubated at room temperature overnight and then washed and redispersed in 4 mM SDS solution. The resulting polymer thickness is around 20 nm after incubation at room temperature overnight and can be controlled by adjusting the amount of monomer in each coating procedure or by repeating the whole coating process to deposit further layers.

### Preparation of NPoM

Atomically smooth planar gold substrates are prepared by the template-stripping method as reported elsewhere.^53,54^ First, 100 nm-thick Au is evaporated onto a silicon wafer at a growth rate of 0.1 nm/s using an e-beam evaporator (Lesker LEV). Then, small pieces (*ca*. 1 cm^2^) of the bare Si wafer are glued on with epoxy and peeled off together with the Au layer. In the end, polymer-coated Au NPs are deposited by drop-casting the colloidal solution onto the Au substrates for 5 min, followed by distilled water rinsing and nitrogen gas drying. Before measurement, the samples are immersed in deionized water overnight to eliminate excess surfactants.

### Electrochemical Cell

A fluid chamber is created by adhering two clean glass coverslips with a stack of center-removed square double-sided tape and filled with electrolyte solution (SI Figure S1). The electrolyte used for Au@PANI and Au@PPy is 0.5 M NaCl in 10 mM HCl, and for Au@PEDOT is 0.5 M NaCl aqueous solution. A low pH environment helps PANI and PPy mitigate the occurrence of irreversible overoxidation.^55,56^ The sample is sandwiched between the coverslips and immersed into the electrolyte. The Au layer substrate is used as the working electrode; the bare area is wired to a potentiostat (Autolab PGSTAT204, Metrohm). A Ag/AgCl reference electrode (3 M KCl, eDAQ ET072, Green Leaf Scientific) and a platinum mesh counter electrode (Alfa Aeser) are inserted into the electrolyte, together with the Au working electrode to form a three-electrode configuration (SI Figure S1).

### Scanning Electron Microscopic Analysis

Scanning electron microscopic (SEM) images of the samples are obtained using a LEO 1530VP (Zeiss) at an accelerating voltage of 10 kV.

### Optical Imaging and Spectroscopy

Optical dark-field (DF) images and spectra of samples are acquired using a charge-coupled-device camera (Infinity 2) and spectrometer (Ocean Optics QE65000) with numerical aperture (NA) 0.8 100× objectives (Olympus LMPLFLN) in a customized microscope (Olympus BX51). A halogen lamp is used as the white light source. SERS measurements are recorded using the same microscope coupled to a 633 nm laser at 100 μW with 1s integration time, and a 0.9 NA Olympus LWD objective for both excitation and collection. Spectra are recorded by an Andor Newton EMCCD camera coupled to a Horiba Triax 320 spectrometer.

### Numerical Simulation

The electromagnetic response of the NPoM is simulated by finite-difference time-domain (FDTD) calculation software (Lumerical Solutions). The Au nanosphere surrounded with the spherical polymer shell is put onto a gold thin rectangular layer with specific thickness. The light is illuminated as a plane wave with desired polarization. The optical properties of gold and polymers are taken from the literature.^17−20^ To simplify the computation, the refractive index of the whole surrounding environment is set to *n* = 1.33 assuming water.

### DFT Calculation

Polymers were modeled as a tetramer repeat unit to calculate the Raman spectra because only small changes in the HOMO and LUMO energy occur when using more repeat units.^57^ Gas phase geometry optimizations and Raman frequency calculations were performed with no symmetry restrictions. B3LYP hybrid functional, def2svp basis set, and Grimme’s D3 dispersion correction with Becke–Johnson damping were used.^58^ To enhance the accuracy of calculations, the UltraFine integration grid was used. For PANI and PPy, TCheck was used to attempt to read a guess from the checkpoint file and to generate another one if necessary. The molecular geometry, charge, multiplicity, and title were read from the checkpoint file. If the first-order convergence method did not converge, it was switched to the second-order convergence method halfway through. For PANI, while complex proton-coupled electron transfer is involved in redox, calculations were able to confirm the protonation states at pH 2 (SI Figure S7c). For PEDOT, to obtain best-fit with experimental SERS for PEDOT, interaction with a gold atom is necessary in calculations (SI Figure S9), revealing that PEDOT provides coordination sites which are distinctive from the other two polymers. Tight convergence was used. The calculated SERS spectra were frequency scaled by factors of 0.94, 0.95, and 0.97 respectively for P^0^, P^1+^, and P^2+^. Slightly different scaling factors were used because the difference in electron delocalization was not fully reflected in the calculations. All DFT calculations were carried out with the Gaussian 09 program package.^59^

## Data Availability

All data for used for the figures is available upon reasonable request from the corresponding author.

## Supporting Information Available

Schematic of the electrochemical cell; FDTD simulations of different *e*NPoMs; FDTD simulations of different nanostructure configurations; color dynamics comparison of different *e*NPoMs; schematics of non-Faradaic capacitive current and scan rate dependence measurement; electroanalysis of polymers; resonance structures and redox of different polymers; assignment of characteristic Raman bands; further analysis of SERS dynamics during redox tracking; PEDOT tetramer-Au structure used in DFT for optimized SERS spectra and calculated UV–vis absorption spectra for PANI, PEDOT, and PPy (PDF)
