# Supplementary material for: In-Situ Spectro-Electrochemistry of Conductive Polymers Using Plasmonics to Reveal Doping Mechanisms
Source: ACS Nano. 2022 Dec 5;16(12):21120–8. doi: 10.1021/acsnano.2c09081 (PMC9798863; doi:10.1021/acsnano.2c09081)
Supplement: Supplementary file 1 — nn2c09081_si_001.pdf [file nn2c09081_si_001.pdf]

Supporting Information for

In-situ Spectro-electrochemistry of Conductive Polymers Using Plasmonics to Reveal Doping Mechanisms

*Jialong Peng, [†#] Qianqi Lin, [†#] Tamás Földes, [‡] Hyeon-Ho Jeong, [†] Yuling Xiong, [†] Charalampos Pitsalidis, [§] George G. Malliaras [‖], Edina Rosta, [‡] Jeremy J. Baumberg[†]**

[†] NanoPhotonics Centre, Cavendish Laboratory, Department of Physics, University of Cambridge, Cambridge CB30HE, UK

[‡] Department of Physics and Astronomy, University College London, London WC1E 6BT, UK

[§] Department of Chemical Engineering and Biotechnology, University of Cambridge, Cambridge CB30AS, UK

[‖] Electrical Engineering Division, Department of Engineering, University of Cambridge, Cambridge CB30FA, UK

Corresponding Author

*Email: jjb12@cam.ac.uk.

Present Addresses

Jialong Peng: College of Advanced Interdisciplinary Studies and Hunan Provincial Key Laboratory of Novel Nano-Optoelectronic Information Materials and Devices, National University of Defense Technology, Changsha 410073, China

Qianqi Lin: Hybrid Materials for Opto-Electronics Group, Department of Molecules and Materials, MESA+ Institute for Nanotechnology and Center for Brain-Inspired Nano Systems, Faculty of Science and Technology, University of Twente, 7500 AE Enschede, The Netherlands

Hyeon-Ho Jeong: School of Electrical Engineering and Computer Science, Gwangju Institute of Science and Technology, Gwangju 61005, Republic of Korea

Charalampos Pitsalidis: Department of Physics and Healthcare Engineering Innovation Center (HEIC), Khalifa University of Science and Technology, P. O. Box 127788, Abu Dhabi, UAE.

#These authors contributed equally.

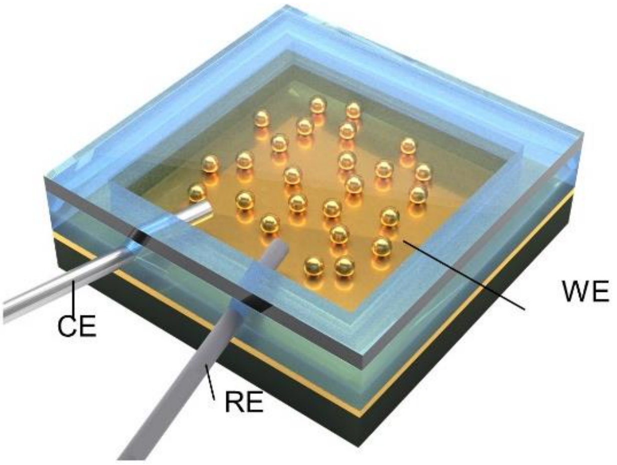

Figure S1. Schematic of the electrochemical cell. The reference electrode (RE) is Ag/AgCl, the counter electrode (CE) is Pt, and the Au layer forms the working electrode (WE).

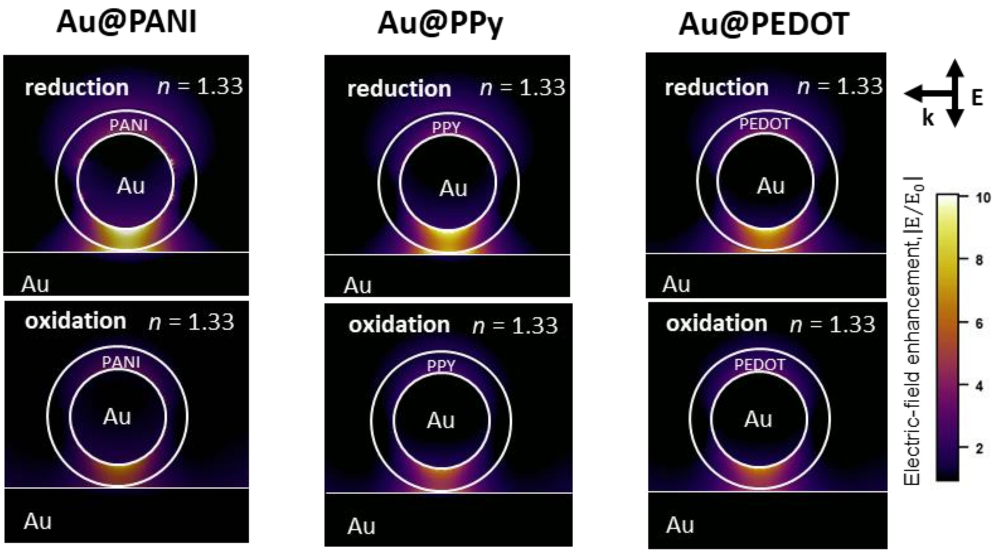

Figure S2. Finite Difference Time Domain (FDTD) simulations of different *e*NPoMs. Optical near-field enhancements of 80 nm diameter Au NPs coated with a 20 nm polymer shell layer (from left to right, Au@ PANI, Au@ PPy and Au@PEDOT; upper panel for reduced state P^0, bottom panel for oxidized state P^{2+}). See Figure1c for corresponding simulated optical scattering and absorption spectra.

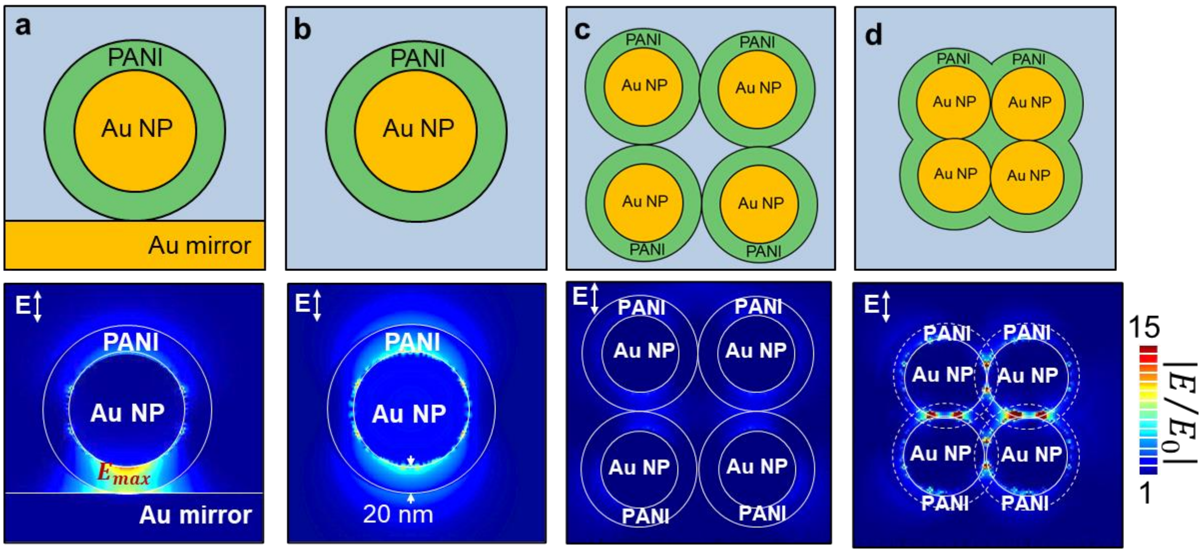

Figure S3. Comparison of different nanostructure configurations in FDTD simulations. Schematics (top panel) and corresponding optical near-field enhancements (bottom panel) of (**a**) *e*NPoM construct, with consistent gap field enhancements in the practical implementation, (**b**) single isolated Au@PANI, (**c**) Au@PANI tetramer, (**d**) Au tetramer aggregate@PANI. The latter two show enhancements vary in different parts of an aggregate, as seen in experiments. The thickness of PANI shell layer is 20 nm and the polymer is set at reduced state. The refractive index of the whole surrounding environment is set to n = 1.33 assuming water for all cases.

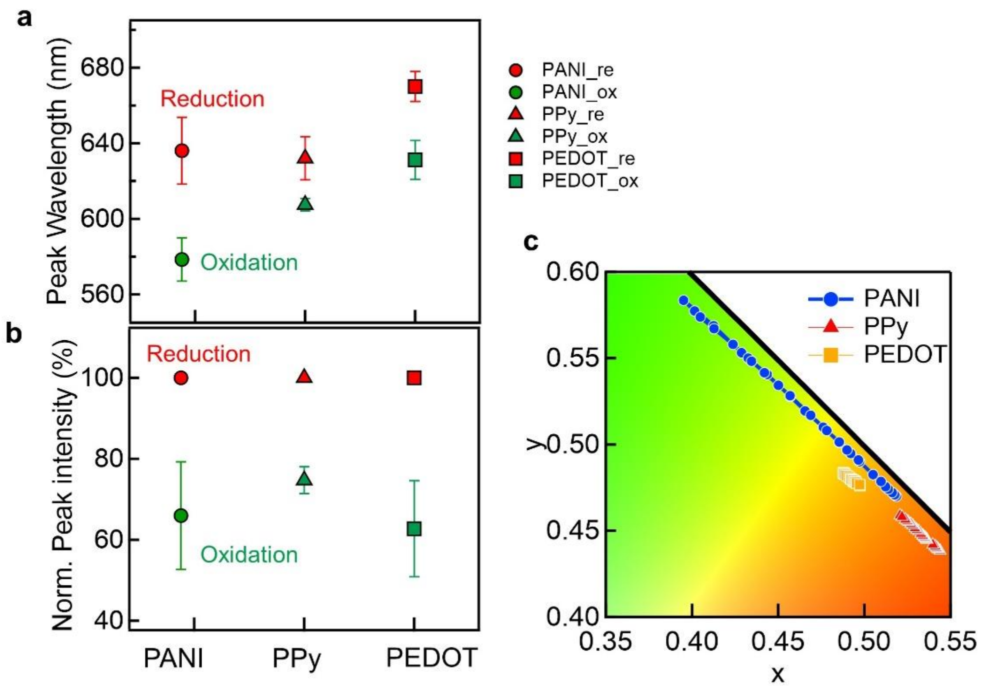

Figure S4. Color dynamics comparison of *e*NPoMs made from Au@PANI, Au@PPy and Au@PEDOT. (**a**) Experimental optical tuning and (**b**) corresponding intensity switching of *e*NPoMs. (**c**) Associated color gamut plots (CIE 1931 chromaticity). The color dynamics of Au@PPy mainly reside in the reddish region and Au@PEDOT lies in the yellowish region, while Au@PANI spans from red and yellow, to green.

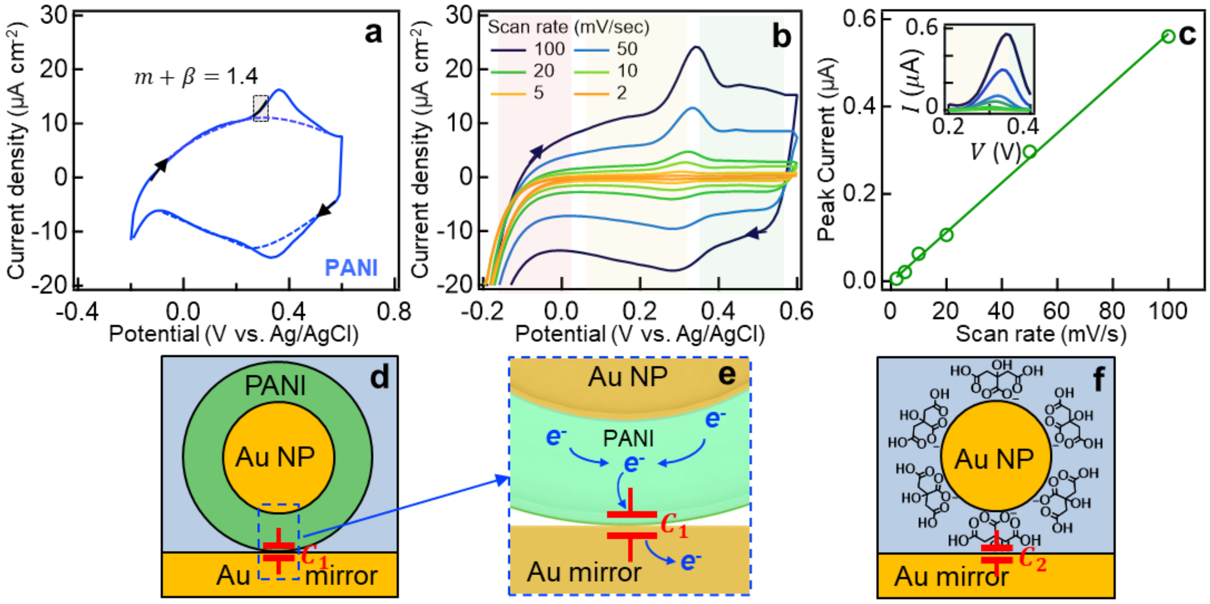

Figure S5. Non-Faradaic capacitive current and scan rate dependence. (**a**) Cyclic voltammetry (CV) of Au@PANI with baseline fit to show background non-Faradaic current from the capacitance C_1 between PANI shell and Au mirror, as shown in (**d**,**e**). This capacitance arises for surface modified electrodes, and the large capacitive current in PANI is consistent with its lower conductivity compared to PEDOT and PPy. (**b**) The non-Faradaic capacitive current is more obvious when CV from different scan rates are overlaid. (**c**) After subtracting the background non-Faradaic current, the peak current vs scan rate is linear, confirming the surface absorption behavior of PANI. (**f**) AuNPs are citrate capped, hence possess a different capacitance C_2 between ligands and the Au mirror. It is thus not possible to experimentally quantify the capacitive current in the *e*NPoM system, hence a baseline fit is used in a.

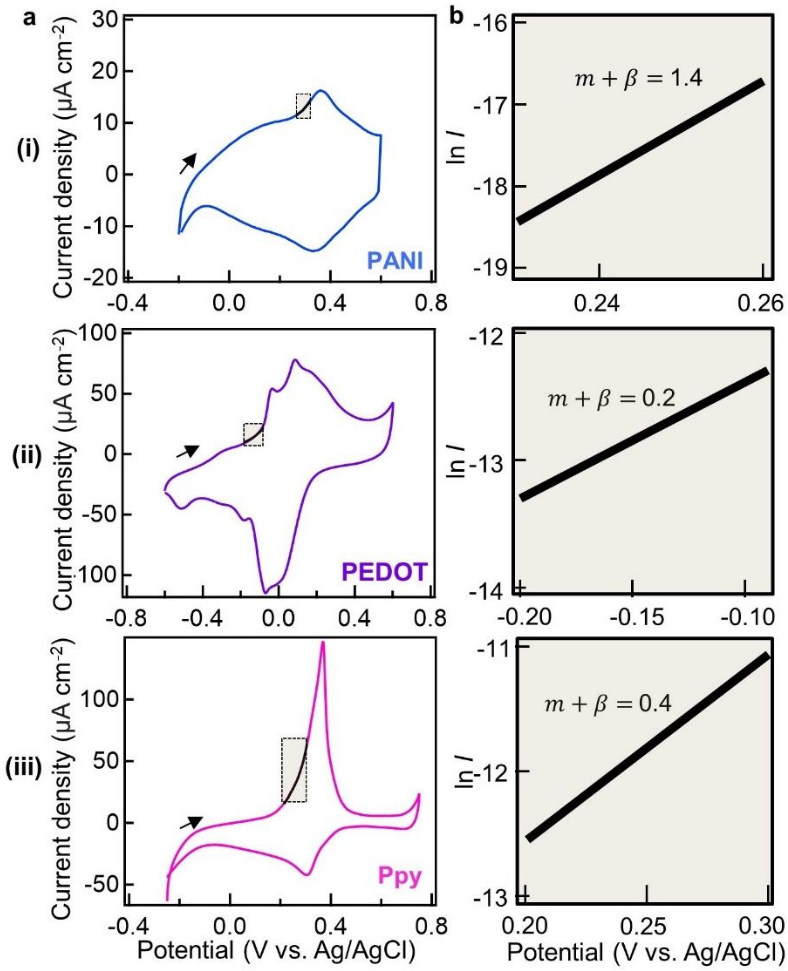

Figure S6. Electroanalysis of polymers. (**a**) Cyclic voltammetry (CV) at a scan rate of 50 mV s^{-1}. (**b**) Tafel plots with apparent transfer coefficients ($m + \beta$) ranging from 0 to 2, showing the transition state is reactant-like (P^0) or produce-like (P^{2+}). Current is extracted after subtracting background non-Faradaic capacitive current, as discussed in Figure S5. (i) PEDOT, (ii) PPy (iii) PANI.

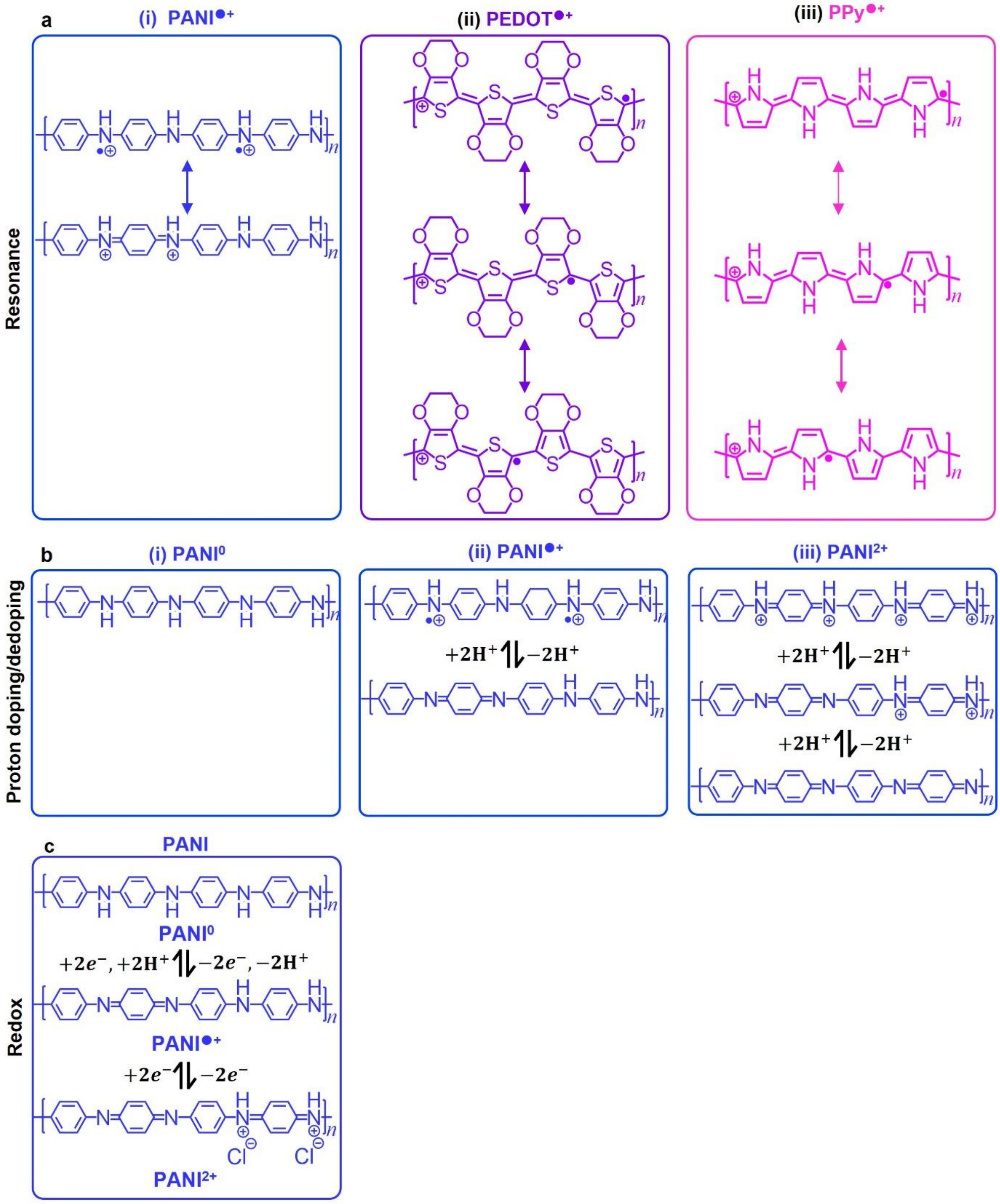

Figure S7. Electronic states and redox. (**a**) Resonance structures of polarons $P^{\bullet+}$. (**b**) Delocalized electronic states along backbone from proton doping and dedoping of PANI. Such conjugated backbone is not in $PANI^0$, but is in $PANI^{\bullet+}$ and $PANI^{2+}$ upon oxidation. There is no similar delocalization in PEDOT or PPy. (**c**) Proton-coupled electron transfer for PANI redox at pH 2, determined by DFT.

Table S1. Assignment of characteristic Raman bands for PANI, PEDOT, and PPy.

Polymers	Oxidation state	Vibrational modes	Wavenumbers (cm^{-1})
PANI[1–3]	0, 1+, 2+	δ(C-H) in-plane	1180 → 1160
	1+	v(C~N+•)	1340
	2+	v(C=N)	1480
	2+	v(C=C) in quinone rings	1580
	0, 1+, 2+	v(C-C) in benzene rings	1620
PEDOT[4–6]	0, 1+, 2+	v(C–O–C) asymmetric	1085
	0	v(C_β-$C_{\beta'}$)	1340
	0 → 2+	v(C_α=C_β) symmetric	1403 → 1427
	0 → 2+	v(C_α=C_β) asymmetric	1527 → 1507, 1541
PPy[7–9]	2+	Ring deformation associated with oxidation	930
	0	Ring deformation associated with reduction	970
	0, 1+, 2+	δ(C-H) in-plane	1040
	0 → 2+	v(C=C) backbone	1545 → 1585

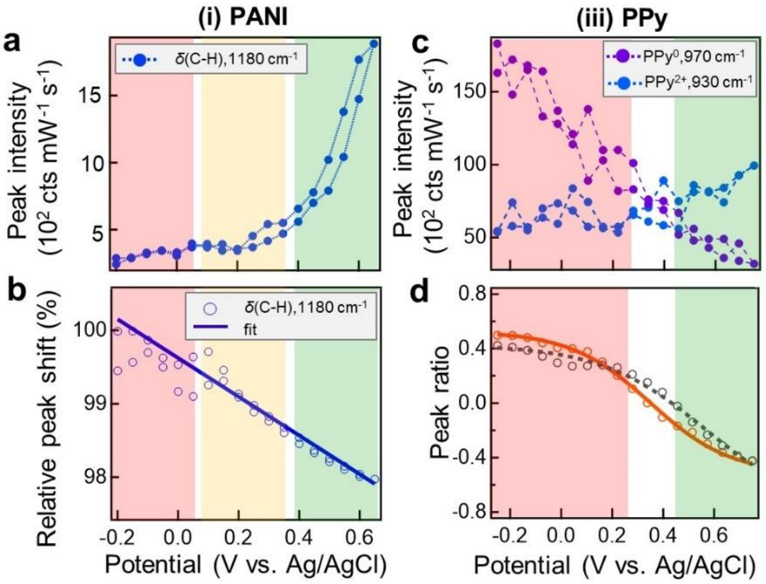

Figure S8. Further analysis of SERS dynamics during redox tracking.

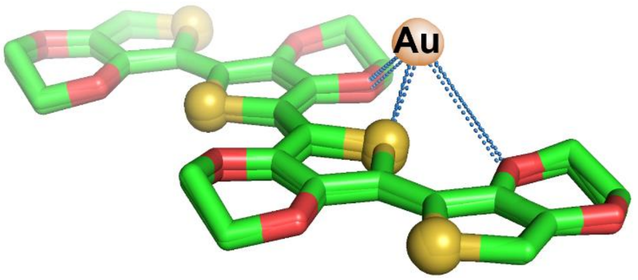

Figure S9. PEDOT tetramer-Au structure used in DFT for optimized SERS spectra.

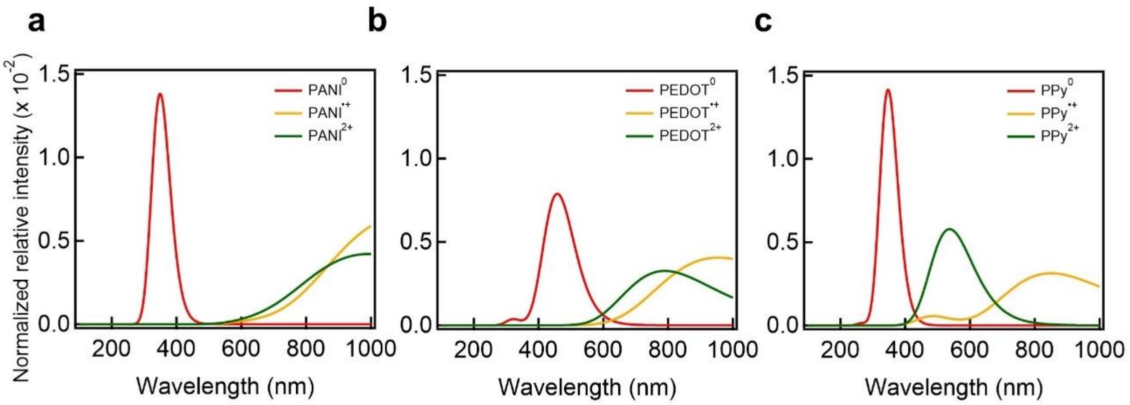

Figure S10. Calculated UV-VIS absorption spectra for PANI, PEDOT, and PPy.

REFERENCES

(1) Mažeikienė, R.; Niaura, G.; Malinauskas, A. Study of Redox and Protonation Processes of Polyaniline by the Differential Multiwavelength Raman Spectroelectrochemistry. *Spectrochim. Acta - Part A Mol. Biomol. Spectrosc.* **2019**, *30*(4), 219-226.

(2) Mažeikienė, R.; Niaura, G.; Malinauskas, A. Raman Spectroelectrochemical Study of Polyaniline at UV, Blue, and Green Laser Line Excitation in Solutions of Different PH. *Synth. Met.* **2018**, *243*, 97–106.

(3) Bernard, M. C.; Hugot-Le Goff, A. Quantitative Characterization of Polyaniline Films Using Raman Spectroscopy. I: Polaron Lattice and Bipolaron. *Electrochim. Acta* **2006**, *52* (2), 595–603.

(4) Savva, A.; Wustoni, S.; Inal, S. Ionic-to-Electronic Coupling Efficiency in PEDOT:PSS Films Operated in Aqueous Electrolytes. *J. Mater. Chem. C* **2018**, *6* (44), 12023–12030.

(5) Garreau, S.; Louarn, G.; Buisson, J. P.; Froyer, G.; Lefrant, S. In Situ Spectroelectrochemical Raman Studies of Poly(3,4-Ethylenedioxythiophene) (PEDT). *Macromolecules* **1999**, *32* (20), 6807–6812.

(6) Kulandaivalu, S.; Zainal, Z.; Sulaiman, Y. Influence of Monomer Concentration on the Morphologies and Electrochemical Properties of PEDOT, PANI, and PPy Prepared from Aqueous Solution. *Int. J. Polym. Sci.* **2016**, *2016*, 1–12.

(7) Hou, Y.; Zhang, L.; Chen, L. Y.; Liu, P.; Hirata, A.; Chen, M. W. Raman Characterization of Pseudocapacitive Behavior of Polypyrrole on Nanoporous Gold. *Phys. Chem. Chem. Phys.* **2014**, *16* (8), 3523–3528.

(8) Santos, M. J. L.; Brolo, A. G.; Girotto, E. M. Study of Polaron and Bipolaron States in Polypyrrole by in Situ Raman Spectroelectrochemistry. *Electrochim. Acta* **2007**, *52* (20), 6141–6145.

(9) Liu, Y. C.; Hwang, B. J.; Jian, W. J.; Santhanam, R. In Situ Cyclic Voltammetry-Surface-Enhanced Raman Spectroscopy: Studies on the Doping-Undoping of Polypyrrole Film. *Thin Solid Films* **2000**, *374* (1), 85–91.
